# Protein homeostasis and inter-organellar signaling under salt stress

**DOI:** 10.1080/15592324.2026.2744693

**Published:** 2026-10-07

**Authors:** Susmita Das, Yamshi Arif, Hesam Mousavi, Henrik Aronsson, Nadia Bazihizina, Mohammad Sarraf

**Affiliations:** a Independent Researcher, Kolkata, India; b Department of Botany, Aligarh Muslim University, Aligarh, India; c Faculty of Applied Ecology, Agricultural Science, and Biotechnology, University of Inland Norway, Elverum, Norway; d Department of Biological and Environmental Sciences, University of Gothenburg, Gothenburg, Sweden; e Department of Biology, Università degli Studi di Firenze, Florence, Italy; f Department of Horticultural Science, Faculty of Agriculture, Shahid Chamran University of Ahvaz, Ahvaz, Iran

**Keywords:** Salt stress, protein homeostasis, inter-organellar signaling, unfolded protein response, post-translational modification, gasotransmitter signaling

## Abstract

Salinity is a major abiotic constraint on plant growth, productivity, and global food security. It disrupts cellular protein homeostasis (proteostasis) at every step, from synthesis to degradation. Plant survival under salt stress therefore depends on the coordinated regulation of protein synthesis, folding, post-translational modification, trafficking, quality control, and turnover. The individual contributions of the nucleus, ribosomes, endoplasmic reticulum (ER), and Golgi apparatus to salinity responses are increasingly well described, but how these compartments communicate remains far less understood. This review synthesizes current evidence on this inter-organellar signaling network, addressing four questions: how salinity-induced osmotic, ionic, and oxidative stress reprograms nuclear transcription and chromatin state; how ribosomes sustain selective translation of protective proteins while clearing stalled or damaged translational products; how the ER expands its folding capacity through the unfolded protein response and removes terminally misfolded proteins by ER-associated degradation; and how the Golgi sustains glycosylation, sorting, and secretion of the transporters needed for ion and osmotic homeostasis. Beyond these organelle-level responses, the review discusses two signaling layers that couple them into a single circuit: post-translational modifications (phosphorylation, ubiquitination, SUMOylation, and redox- and nitrosative/persulfidation-based marks) and phytohormone/gasotransmitter signals, including abscisic acid, nitric oxide, hydrogen sulfide, carbon monoxide, and melatonin. By framing proteostasis as an emergent property of nucleus-ribosome-ER-Golgi coordination rather than of any single compartment, this review identifies specific inter-organellar signaling nodes as candidate targets for breeding or engineering salinity-resilient crops.

## Graphical abstract

**Figure:**
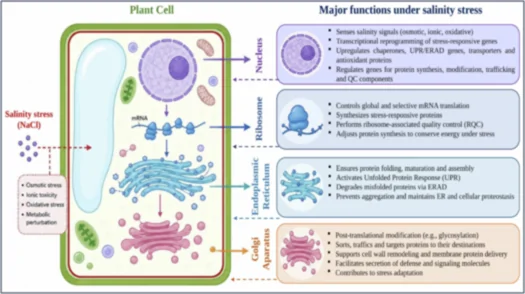

Overview of the four core organelles of the plant secretory and translational machinery - nucleus, ribosome, endoplasmic reticulum, and Golgi apparatus - and their principal functions in maintaining protein homeostasis under salt stress.

## 1. Introduction

Soil salinity is a major abiotic constraint threatening agricultural productivity and food security worldwide.^1^ ^,^ ^2^ Nearly 20% of global arable land and about 50% of irrigated land are affected by salinity, a proportion expected to rise under climate change, sea-level rise, and unsustainable irrigation.^3^ ^,^ ^4^ FAO’s Global Map of Salt-Affected Soils (GSASmap) estimates that more than 833 million hectares (~8.7% of global land) were salt-affected at launch, with subsequent FAO assessments, based on expanded country-level mapping coverage, placing the affected area above 1,380 million hectares (~10.7% of global land).^5^

Salinization degrades soil structure and fertility and is increasingly driven by anthropogenic factors such as poor irrigation management, insufficient drainage, and the use of marginal-quality water.^6^ ^,^ ^7^ Salinity stress arises primarily from excessive accumulation of soluble ions, notably Na^+^, Ca^2^^+^, K^+^, and Mg^2^^+^, in the soil solution.^8^ ^,^ ^9^ At high concentrations, these ions lower soil water potential and impose combined osmotic and ionic stress on plants. Among the salts responsible, sodium chloride (NaCl), calcium chloride (CaCl_₂_), and magnesium chloride (MgCl_₂_) are the most common; NaCl is particularly deleterious because of its pronounced effects on ion toxicity, membrane integrity, and nutrient imbalance relative to calcium-based salts.^10^ ^,^ ^11^

At the protein level, salinity disrupts structure, function, and turnover through combined osmotic, ionic, and oxidative effects. Osmotic stress reduces cellular water availability, stripping proteins of the hydration shell required for their native conformation and promoting misfolding, aggregation, or denaturation.^12^ ^,^ ^13^ Ionic toxicity, driven mainly by excess Na^+^ and Cl^−^, disturbs electrostatic interactions such as salt bridges and hydrogen bonds that stabilize protein structure,^14^ and Na^+^ can displace K^+^ at enzyme-activation sites, further impairing catalytic function.^15^ Downstream, salinity suppresses transcription and translation of many housekeeping proteins,^16^ disrupts chaperone-assisted folding,^17^ and alters phosphorylation, glycosylation, and other post-translational modifications that govern protein stability and signaling.^18^ ^,^ ^19^ The resulting imbalance between synthesis of stress-protective proteins (HSPs, LEA proteins, osmotin) and degradation of damaged proteins is more severe in salt-sensitive genotypes than in tolerant ones, underscoring that proteostatic resilience, not simply protein abundance, distinguishes tolerance from sensitivity.^20-22^

Our previous work highlighted that salinity tolerance depends on coordinated interactions among cellular protectors, including the cell wall, plasma membrane, vacuole, and peroxisome, together with their functional crosstalk with intracellular organelles such as the nucleus and endoplasmic reticulum (ER).^23^ Building on this cellular framework, this review focuses on how salinity-induced stress is transmitted to the protein-synthesis, folding, processing, and quality-control machinery across the nucleus-ribosome-ER-Golgi continuum.

These protein-level disturbances originate in, and are coordinated across, four interconnected organelles. In the nucleus, salt-induced osmotic and ionic stress condenses chromatin and increases ROS-mediated DNA damage,^24^ altering expression of stress-responsive transcription factor families, including DREB and NAC,^25^ ^,^ ^26^ MYB,^27^ WRKY,^28^ ^,^ ^29^ and bZIP and bHLH,^30^ ^,^ ^31^ together with epigenetic marks that encode stress memory.^32-34^ Ribosomes, the protein-synthesis machinery, show impaired rRNA processing and assembly under ionic and oxidative stress,^35^ ^,^ ^36^ reduced translation initiation and overall protein output,^37^ ^,^ ^38^ yet selectively maintain translation of stress-protective mRNAs.^39^ ^,^ ^40^

The ER, responsible for folding and maturing secretory and membrane proteins, accumulates misfolded proteins under salinity, triggering ER stress and the unfolded protein response (UPR), which upregulates chaperones such as BiP and calnexin while attenuating global translation^41^ ^,^ ^42^; when this capacity is exceeded, ER-associated degradation (ERAD) or programmed cell death can follow.^43^ ^,^ ^44^ The Golgi apparatus, which glycosylates, sorts, and traffics proteins destined for the cell wall and plasma membrane, shows disrupted vesicular transport and cell-wall polysaccharide secretion under salt-induced membrane destabilization.^45-47^

Notably, salinity’s impact is not confined to these four compartments. Photosynthesis, which occurs in the plastids, is strongly affected by ion toxicity, and this depends critically on nuclear–plastid protein exchange: an estimated 3,000-4,000 nuclear-encoded proteins are imported into plastids to sustain their proteome.^48^ Salt stress retards chlorophyll accumulation in wheat (*Triticum aestivum*) by limiting protochlorophyllide formation via the nuclear-encoded enzyme protochlorophyllide oxidoreductase (POR)^49^ and induces swollen prothylakoids and altered prolamellar-body remodeling in dark-grown wheat leaves,^50^ while comparative proteomics of salt-stressed wheat chloroplasts shows extensive remodeling of the photosynthetic and protein-folding proteome.^51^ These findings illustrate that nuclear transcriptional and translational reprogramming under salinity extends beyond the four core organelles discussed here to shape the plastid proteostasis as well.

Despite this progress, most studies address individual organelles or pathways in isolation, and a synthesis linking the nucleus, ribosome, ER, and Golgi within a single mechanistic framework of salinity-induced proteostasis is still lacking. This review therefore integrates recent evidence on how salinity disrupts, and plants restore, proteostasis through coordinated dysfunction and repair across the nucleus-ribosome-ER-Golgi continuum, with particular emphasis on the molecular crosstalk, signaling pathways, and quality-control strategies that link these compartments, and on the biotechnological opportunities this integrated view opens for improving salinity tolerance in crops.

## 2. Structure, function, and salinity-responsive dynamics of the nucleus, ribosomes, endoplasmic reticulum, and Golgi apparatus in plants

In unstressed plant cells, protein biogenesis and trafficking are coordinated through an interconnected pathway involving the nucleus, ribosomes, ER, and Golgi apparatus. Gene transcription occurs in the nucleus, followed by the export of mRNAs into the cytoplasm. The nucleolus facilitates the assembly of ribosomal subunits, which mediate cytosolic translation. Nascent secretory and membrane proteins are co-translationally translocated into the ER, where molecular chaperones and folding machinery, including BiP, protein disulfide isomerase (PDI), and the calnexin/calreticulin (CNX/CRT) cycle, facilitate protein folding and quality control. Properly folded proteins are subsequently transported to the Golgi apparatus for further glycosylation, sorting, and vesicular trafficking to their final destinations, including the plasma membrane, cell wall, vacuole, and extracellular space (Figure 1). The organelle-specific responses to salinity discussed in the following subsections can therefore be understood as stress-induced perturbations of, and adaptive adjustments within, this interconnected protein biogenesis and trafficking pathway.

**Figure 1.**
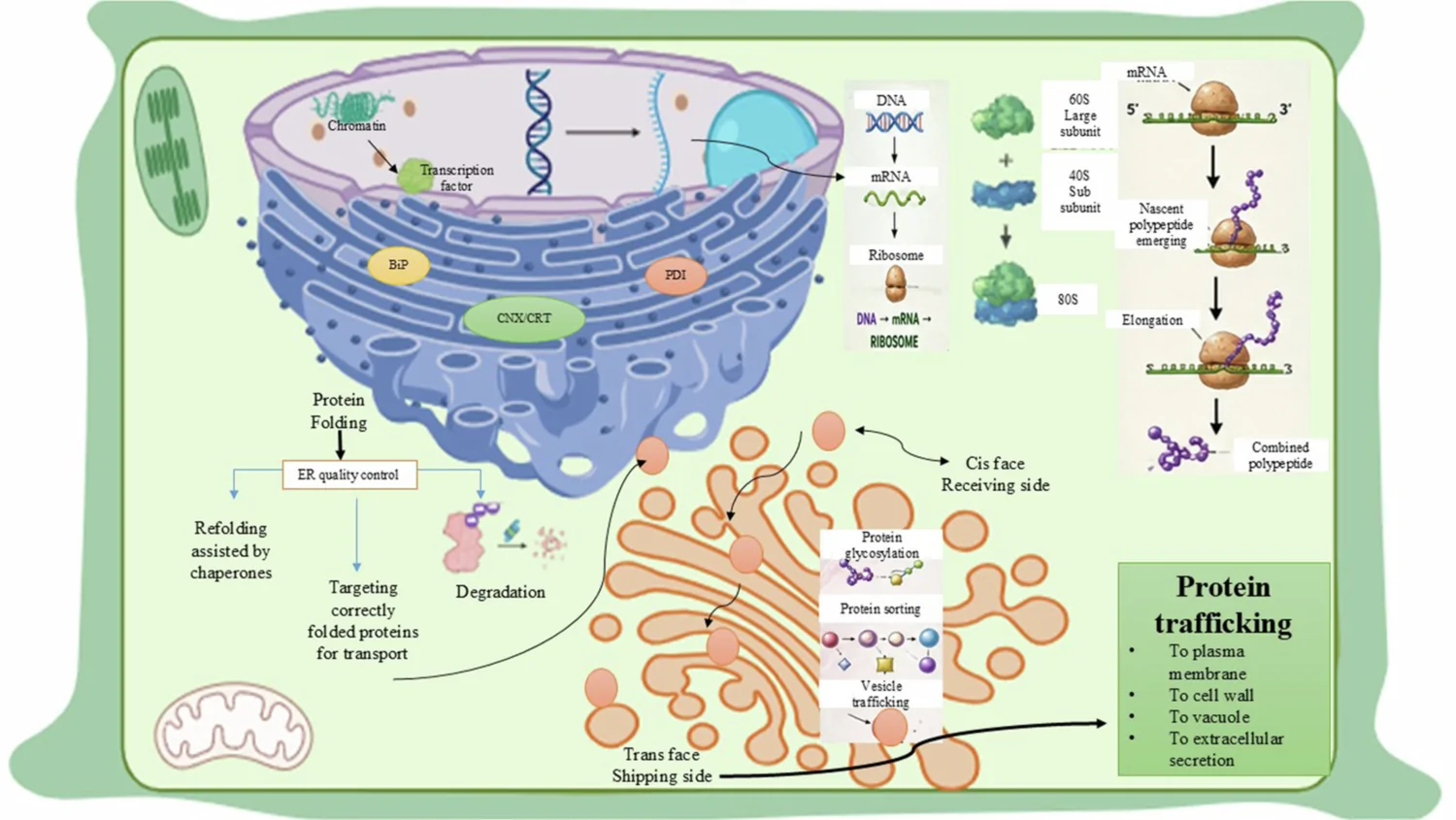
The nuclear-to-Golgi protein synthesis and secretory pathway under normal conditions: transcription and nuclear export, translation by cytosolic ribosomes, co-translational translocation into the endoplasmic reticulum with chaperone-assisted folding (BiP, PDI, CNX/CRT) and ER quality control, and Golgi-mediated glycosylation, sorting, and vesicular trafficking to the plasma membrane, cell wall, vacuole, and extracellular space.

### 2.1. Nucleus

#### 2.1.1. Nuclear architecture as a stress-signaling hub

The nucleus is the plant cell's genetic and regulatory center, coordinating transcriptional reprogramming and stress-responsive signaling under salinity.^52-54^ Salinity-induced changes in chromatin organization and epigenetic modifications, including DNA methylation and histone acetylation, modulate stress-responsive gene expression and thereby influence cellular proteostasis.^55^ In parallel, the nucleolus regulates rRNA transcription, processing, and ribosome biogenesis, linking nuclear stress responses to protein-synthesis capacity. Because approximately 3,000-4,000 nuclear-encoded proteins are trafficked to plastids to sustain photosynthetic function,^48^ nuclear transcriptional reprogramming under salinity also influences plastid proteostasis.

#### 2.1.2. Nuclear transport and organelle connectivity

Nuclear transport provides an additional regulatory interface during salt stress. Nuclear pore complexes (NPCs) control the selective exchange of proteins, RNAs, and ribonucleoprotein complexes between the nucleus and cytoplasm,^56-58^ thereby enabling rapid redistribution of transcription factors and other regulatory proteins in response to stress. The outer membrane of the nuclear envelope is continuous with the ER and further provides structural and functional connectivity between nuclear regulation and ER-mediated protein homeostasis.^59-62^ Extensive signaling between the nucleus, chloroplasts, mitochondria, ER, and Golgi positions the nucleus as a central hub coordinating transcriptional responses, protein synthesis, and organellar adaptation to salinity.^63-65^

#### 2.1.3. Chromatin remodeling and transcriptional reprogramming

Excess Na^+^ and Cl^−^, combined with osmotic imbalance and ROS accumulation, disrupt nuclear organization and gene expression at several linked levels.^29^ ^,^ ^66^ ^,^ ^67^ Mechanistically, osmotically driven loss of turgor reduces nuclear volume and deforms the nuclear envelope. In parallel, chromatin-remodeling complexes reposition nucleosomes: stress-responsive promoters open while growth-related loci are compacted. This redistribution gives transcription factors rapid access to salt-responsive genes that govern ion homeostasis, osmolyte biosynthesis, ROS scavenging, and hormone signaling.^68-72^ This chromatin restructuring is accompanied, and partly driven, by specific histone marks: increased H3K9 and H3K27 acetylation is generally associated with activation of salt-responsive genes, whereas shifts in H3K4me3 and H3K27me3 modulate the balance between transcriptional activation and repression.^34^ ^,^ ^73-76^ Salinity further alters CG, CHG, and CHH DNA methylation, affecting transposable-element activity and genome stability and contributing to transgenerational stress memory.^77^ Downstream, stress-responsive transcription factors of the DREB, NAC, WRKY, MYB, and bZIP families accumulate and regulate genes controlling osmotic adjustment, ion transport, antioxidant defense, and hormonal signaling.^26^ ^,^ ^78^ ^,^ ^79^

Whether the chromatin machinery is constitutively primed or induced de novo may distinguish salt-tolerant from salt-sensitive taxa. In the halophyte saltwater cress (*Eutrema salsugineum*), salt-responsive genes are enriched among transcripts that already use distal, stress-adapted polyadenylation sites under non-saline conditions. Therefore, relatively few loci require further reprogramming after salt stress. In contrast, the salt-sensitive glycophytic relative Arabidopsis (*Arabidopsis thaliana*) shows a much larger salt-induced shift in alternative polyadenylation. This pattern suggests that its transcriptional and chromatin machinery undergoes substantial reconfiguration after stress onset rather than being pre-set for salt stress.^80^ A comparable sensitivity phenotype has been traced to a single nucleolar protein in Arabidopsis: loss of the Pumilio-domain protein APUM23 destabilizes rRNA processing and triggers ABA-hypersensitive salt intolerance, showing that the same nucleolar-ribosome biogenesis axis discussed below can, when disrupted, convert a normally salt-tolerant genotype into a hypersensitive one.^81^ Such glycophyte-halophyte and mutant-wild-type contrasts indicate that the transcription factor families and chromatin marks described above are necessary but not sufficient for tolerance; their pre-stress accessibility and speed of deployment are what separate tolerant from sensitive genotypes.

#### 2.1.4. Post-transcriptional control and the nucleolus-ribosome interface

Salinity also reshapes post-transcriptional RNA metabolism: alternative splicing increases markedly, generating multiple transcript isoforms from single genes and expanding the stress-response proteome,^82^ while RNA-binding proteins and splicing factors are upregulated to safeguard accurate processing, editing, export, and turnover of stress transcripts.^35^ Because the nucleolus itself is salt-sensitive, its morphology and rRNA transcription and processing are altered under saline conditions.^83^ ^,^ ^84^ ROS generated during salt stress also cause direct oxidative damage to nuclear DNA, RNA, and protein, which base-excision, nucleotide-excision, and homologous-recombination repair pathways counteract; ROS also serves as a stress-signaling cue.^85^ ^,^ ^86^

#### 2.1.5. Nuclear proteostasis: damage and restorative quality control

A central question is whether salinity disrupts or maintains nuclear proteostasis. Elevated ROS oxidizes transcription factors, chromatin-associated proteins, RNA-processing enzymes, and DNA-repair proteins, driving misfolding and aggregation. However, plants counter this with robust quality control: HSP70, HSP90, and small HSPs are strongly induced and refold damaged nuclear proteins,^87^ ^,^ ^88^ while nuclear E3 ubiquitin ligases tag irreversibly damaged proteins for 26S-proteasomal removal via the ubiquitin–proteasome system (UPS).^89-91^ RNA quality-control pathways further limit the synthesis of defective transcripts. Together, these mechanisms establish a new proteostatic equilibrium rather than a permanent loss of function; the speed with which this equilibrium is re-established is itself a determinant of salt tolerance, being faster in tolerant genotypes than in sensitive ones.^88^

### 2.2. Ribosomes

#### 2.2.1. Translational reprogramming and selective mRNA usage

Ribosomes are the central machinery of protein synthesis, converting mRNA into proteins through initiation, elongation, termination, and recycling, with initiation being particularly sensitive to stress.^92-94^ Beyond bulk translation, ribosomes regulate proteostasis through selective mRNA translation, preferentially supporting the synthesis of stress-protective proteins such as HSPs, antioxidant enzymes, osmoprotectant-biosynthetic enzymes, and transcription factors while restricting non-essential protein production.^95-98^ Under salinity, disruption of rRNA processing, ribosome biogenesis, or translation can therefore compromise the supply of proteins required for cellular adaptation.

#### 2.2.2. Ribosome biogenesis, specialization, and halophyte-glycophyte contrasts

Plant cytosolic 80S ribosomes are assembled through a nucleolus-dependent pathway, linking nuclear rRNA processing and ribosome biogenesis with cytoplasmic protein synthesis; chloroplasts and mitochondria additionally possess 70S ribosomes for organellar gene expression.^84^ ^,^ ^92^ ^,^ ^99^ The large diversity of plant ribosomal protein paralogues may generate heterogeneous or “specialized” ribosomes with preferences for particular mRNA subsets, potentially enabling stress-specific translational programs.^100^ ^,^ ^101^ This translational flexibility potentially redirects protein synthesis toward protective pathways during salinity stress.

As protein synthesis is highly energy-demanding, salinity-induced oxidative stress can impair rRNA maturation and ribosome assembly, while severe stress may suppress ribosome biogenesis to conserve resources.^12^ ^,^ ^38^ Nevertheless, plants can remodel translation to favor stress-responsive mRNAs over housekeeping transcripts. This selective translation is mediated by mechanisms including uORF-dependent translational control, RNA-binding proteins that regulate mRNA recruitment to initiation complexes, and changes in the activity or availability of translation initiation factors such as eIF2, eIF3, eIF4E, and eIF4G.^38^ ^,^ ^102^ ^,^ ^103^ Consequently, although global translation declines, synthesis of proteins involved in ion homeostasis, osmoprotection, antioxidant defense, chaperone activity, and stress signaling can be preferentially maintained.^104^ ^,^ ^105^

Comparative ribosome profiling further indicates that salt-tolerant rice (*Oryza sativa*) maintains a distinct, more stress-protective translational landscape than salt-sensitive genotypes under comparable salt exposure.^106^ In the extreme halophyte Salicornia (*Salicornia bigelovii)*, the intrinsically disordered RGG-domain protein SALTY binds ribosomes and RNA-containing structures and is required for extreme salinity tolerance, providing a possible molecular explanation for how translation is sustained under conditions that would otherwise be inhibitory.^107^ Whether such specialized, translation-protective domains are a general feature of tolerance or a halophyte-specific innovation is directly testable by comparison with salt-sensitive relatives: in the glycophyte model Arabidopsis, which lacks a SALTY ortholog, ribosome-associated RNA-binding proteins do not confer comparable protection, and salt sensitivity in this species instead traces to loss-of-function of core ribosome-biogenesis regulators such as the nucleolar protein APUM23 rather than to acquisition of a stabilizing domain.^81^ Thus, the halophyte-glycophyte comparison suggests that extreme salinity tolerance may involve the acquisition or functional specialization of additional protective modules, rather than simply a stronger activation of conserved stress responses.

Comparable genotype-dependent differences in translational responses have also been observed within a single crop species. Comparative ribosome profiling of salt-tolerant and salt-sensitive rice cultivars showed that both groups activate salt-responsive translational programs, but tolerant cultivars more strongly redirect ribosome loading toward transcripts associated with antioxidant defense and ion transport under equivalent salt exposure.^106^ This suggests that differences in salt tolerance within a crop may arise not from completely distinct response mechanisms, but from differences in the magnitude and translational prioritization of shared stress-response pathways, which may be relevant to breeding.

#### 2.2.3. Ribosome damage, quality control, and turnover

Salinity-driven ribosome heterogeneity compounds this selectivity: differential incorporation of paralogous ribosomal protein isoforms generates structurally distinct ribosome populations with biased mRNA affinities, adding another regulatory layer to stress translation. The precise structural basis of this specialization remains under active investigation.^37^ ^,^ ^101^ ^,^ ^108^ ^,^ ^109^ ROS accumulation directly damages rRNA and ribosomal proteins, compromising subunit interactions, codon recognition, and elongation fidelity.^92^ ^,^ ^110^ This damage necessitates surveillance: the ribosome-associated quality control (RQC) pathway recognizes stalled ribosomes, dissociates subunits, degrades defective mRNAs, and targets incomplete nascent chains for ubiquitin-mediated proteolysis.^95^ ^,^ ^103^

Selective autophagic turnover of damaged ribosomes (ribophagy) removes persistently defective translational machinery and recycles its components into stress-defense metabolism.^111^ ^,^ ^112^ Molecular chaperones of the HSP70/HSP40 and small-HSP families further protect nascent polypeptides from aggregation as they emerge from stalled or actively translating ribosomes.^113^ Collectively, these mechanisms show that ribosomal proteostasis under salinity is not lost but dynamically re-established through chaperone-assisted folding, RQC, ribophagy, and ubiquitin–proteasome clearance, with the efficiency of this re-establishment distinguishing salt-tolerant from salt-sensitive genotypes.^106^ ^,^ ^114^

### 2.3. Endoplasmic reticulum

#### 2.3.1. ER protein folding and export under normal conditions

The ER is the principal site for the folding and maturation of secretory and membrane proteins. Ribosomes associated with the rough ER (RER) co-translationally translocate nascent proteins into the ER lumen, where chaperones such as BiP, calnexin (CNX), calreticulin (CRT), and protein disulfide isomerases (PDIs) facilitate folding and disulfide-bond formation.^115-118^ The ER also performs *N*-linked glycosylation and quality control, directing irreparably misfolded proteins to ER-associated degradation (ERAD) and subsequent proteasomal degradation.^119-121^

Correctly folded proteins are exported in COPII vesicles to the Golgi, while ER Ca^2^^+^ homeostasis provides an additional link between protein folding and stress signaling.^122^ ^,^ ^123^ Under salinity, increased protein misfolding and disruption of ER homeostasis activate the unfolded protein response (UPR), principally through the ER stress sensor IRE1 and the membrane-associated transcription factors bZIP17 and bZIP28.^124^ ^,^ ^125^ The UPR enhances chaperone capacity, adjusts protein synthesis, and promotes degradation of misfolded proteins to restore ER proteostasis; when ER stress exceeds the adaptive capacity of these systems, prolonged stress can contribute to programmed cell death. Thus, the ER functions as a critical quality-control hub connecting protein synthesis with folding, degradation, trafficking, and stress-responsive signaling.

#### 2.3.2. Salt-induced ER stress and the unfolded protein response

Salinity combines osmotic dehydration, ionic toxicity, and oxidative stress, challenging ER function at multiple levels simultaneously. Osmotic dehydration limits ER expansion and disrupts ER–plasma-membrane contact sites and ER-Golgi vesicular flow,^126^ while salt-induced lipid peroxidation compromises membrane integrity and the organization of ER-resident protein complexes.^127^ At the core of the response, excess synthesis of stress proteins together with oxidative and ionic damage overwhelms the ER’s protein-folding capacity, causing unfolded and misfolded proteins to accumulate, a condition termed ER stress and one of the earliest molecular signatures of salinity-induced cellular dysfunction.^42^ This triggers the UPR: activated IRE1 splices bZIP60 mRNA to generate a transcription factor that migrates to the nucleus and induces genes encoding BiP, CNX, CRT, PDIs, and other folding catalysts, expanding folding capacity while curbing the influx of new protein.^42^ ^,^ ^128^

This is not a generic stress response, several studies show that specific HSP/chaperone inductions confer measurable, mechanism-linked protection against the downstream consequences of salinity. For example, elevated BiP expression is consistently associated with enhanced salinity tolerance across species. This indicates that boosting ER folding capacity is an adaptive strategy in its own right, not merely a passive consequence of stress.^42^ ^,^ ^129^ More broadly, HSP70/HSP90 induction protects against the oxidative component of salinity stress by limiting ROS-driven protein aggregation. This preserves enzymatic and signaling activity that would otherwise be lost to oxidative damage.^87^ ^,^ ^88^

#### 2.3.3. ER redox balance and coupling to ion transport

ER redox balance is itself a casualty and a driver of this process, excess ROS oxidizes protein thiols and folding enzymes, while oxidative protein folding itself generates H₂O₂ as a by-product, so that antioxidant systems, glutathione metabolism, and thiol-disulfide exchange become essential for preserving ER function under salinity.^127^ This redox control is not confined to ER-resident thiols. In tomato (*Solanum lycopersicum*) seedlings, exogenous nitric oxide (NO), supplied as S-nitrosoglutathione, increases endogenous protein S-nitrosylation. It also improves salt tolerance. Blocking NO signaling reverses this protective effect. These findings indicate that reactive-nitrogen-species-based redox modification directly intersects with the thiol-dependent folding chemistry described here.^130^ Glycoprotein maturation, monitored through the calnexin–calreticulin cycle, is frequently impaired, prolonging retention of immature glycoproteins and intensifying ER stress until ERAD clears them via retrotranslocation, ubiquitination, and 26S-proteasomal degradation.^117^ ^,^ ^119^ ^,^ ^121^

Salt-induced shifts in ER Ca^2^^+^ concentration also alter the activity of Ca^2^^+^-dependent chaperones (CRT, CNX) and downstream trafficking and signaling, linking Ca^2^^+^ and protein-folding homeostasis.^123^ Importantly, ER function under salinity extends beyond protein folding to the direct regulation of ion transport. In rice, the ER-localized cytochrome b5 (OsCYB5-2) physically interacts with the plasma-membrane K^+^ transporter OsHAK21. This interaction enhances its K^+^-selective uptake activity during salt stress. It thereby links ER-resident post-translational regulation to whole-cell ion homeostasis.^131^ This provides an example of how ER quality-control and regulatory machinery contributes to salt tolerance beyond classical UPR and ERAD pathways.

### 2.4. Golgi apparatus

#### 2.4.1. Golgi processing and secretory trafficking under normal conditions

The Golgi apparatus is the principal processing, sorting, and trafficking hub of the plant secretory pathway, receiving proteins and other cargo from the ER and modifying them through glycan maturation, O-glycosylation, phosphorylation, sulfation, and proteolytic processing.^132-134^ The trans-Golgi network (TGN) subsequently sorts processed cargo into vesicles destined for the plasma membrane, vacuole, cell wall, or extracellular space.^135^ In plants, the Golgi is also essential for cell-wall biogenesis through the synthesis and secretion of hemicelluloses, pectins, and arabinogalactan proteins, thereby linking protein and polysaccharide trafficking with cell growth and defense.^136^ ^,^ ^137^

Plant Golgi stacks are highly mobile and closely associated with the ER, enabling continuous exchange of secretory cargo.^138^ ^,^ ^139^ This trafficking system depends on the coordinated action of glycosylation enzymes and vesicular transport machinery, including COPI, clathrin, Rab GTPases, SNAREs, tethering factors, and the conserved oligomeric Golgi (COG) complex.^140^ ^,^ ^141^ Disruption of Golgi processing or vesicular trafficking under salinity can therefore compromise the maturation, sorting, and delivery of proteins and cell-wall components, making Golgi function an important downstream component of cellular proteostasis.

#### 2.4.2. Salt-induced disruption of Golgi trafficking

The Golgi serves as an active regulatory hub during salinity adaptation rather than merely functioning as a passive trafficking station. It modulates secretion, vesicle transport, and quality control.^142^ Salt-induced osmotic imbalance and changes in membrane lipid composition can alter Golgi cisternal organization and vesicle dynamics. Oxidative damage can also reduce membrane fluidity. Furthermore, it can directly oxidize glycosyltransferases, SNARE proteins, and Rab GTPases. These proteins are essential for cargo sorting and vesicle formation.^143^ ^,^ ^144^ Vesicle budding, docking, and fusion require the coordinated action of several components. These include COPI/COPII coats, clathrin, Rab GTPases, SNAREs, tethering complexes, and the cytoskeleton. Ionic imbalance and oxidative stress can interfere with each of these trafficking steps. This provides a mechanistic explanation for how salinity causes the trafficking defects described in the plant salinity literature. These defects are not merely generic consequences of stress.

#### 2.4.3. Functional consequences for ion transport across the salt tolerance continuum

The functional consequences of Golgi disruption are substantial and specific. Efficient Golgi trafficking is required to deliver plasma-membrane Na^+^/H^+^ antiporters, K^+^ transporters, aquaporins, and ABA-signaling receptor-like kinases to their sites of action. Disrupted Golgi trafficking can therefore directly impair ion and osmotic homeostasis. This can occur even when the transporters themselves remain structurally intact.^142^ Among these receptor-like kinases, the plasma-membrane protein FERONIA provides an example of the connection between secretory delivery, cell-wall sensing, and salt tolerance. FERONIA functions at the ER-Golgi-plasma-membrane interface. It contains an extracellular malectin-like domain. This domain has the same fold as that found in ER-resident quality-control machinery that binds carbohydrate ligands.

In Arabidopsis, FERONIA is required for roots to sense salt-induced cell-wall softening. It also helps roots resume growth after salt exposure.^145^ The FERONIA-RALF receptor–ligand module is not restricted to glycophytic model plants. A genome-wide survey of the halophytic crop quinoa (*Chenopodium quinoa*) identified 26 FERONIA-family receptor genes and 18 RALF-family ligand genes. Many of these genes showed salt-responsive expression. These findings indicate that the Golgi-trafficked FERONIA–RALF receptor system is also present in a naturally salt-tolerant crop. The system may have been retained and diversified during quinoa’s adaptation to saline environments.^146^ A similar regulatory principle operates at the ER level, where an ER-localized cytochrome b5 modulates OsHAK21 activity and thereby regulates K^+^ uptake.^131^ Together, these findings show that ion homeostasis under salinity depends not only on transporter gene expression but also on their correct secretory delivery and post-translational regulation.^107^ ^,^ ^131^

#### 2.4.4. Cell-wall remodeling and the ER-Golgi quality-control relay

The Golgi also mounts an adaptive, salt-specific response in cell-wall remodeling; transcriptional and biochemical upregulation of Golgi-localized glycosyltransferases and polysaccharide-biosynthetic enzymes alters wall composition to increase rigidity, reduce water loss, and improve mechanical stability under osmotic stress.^45^ ^,^ ^47^ Because the Golgi operates immediately downstream of the ER, disturbances in ER folding propagate directly to it: proteins that escape ER quality control under severe salinity add to the Golgi’s processing burden, but the Golgi itself provides a second checkpoint, retrieving incorrectly glycosylated or improperly processed proteins to the ER for refolding or routing them to vacuolar and ubiquitin-dependent degradation.^143^ This two-tier ER–Golgi quality-control relay, rather than either organelle acting alone, is what allows plants to maintain a functional secretory proteome under sustained salinity.

Across all four compartments, a common trajectory from balance through disruption to compensation can be observed. Under normal conditions, gene regulation, translation, protein folding, and trafficking function as a coordinated pipeline. Salinity stress disrupts these processes sequentially, involving genome instability and altered transcription in the nucleus, reduced translational fidelity at the ribosome, ER stress and protein misfolding, and impaired Golgi trafficking. The cumulative effects of these disturbances lead to system-wide proteostasis imbalance and functional decline (Figure 2). Table 1 summarizes the structural and functional roles of each organelle, the specific disruptions induced by salinity, and the restorative mechanisms that contribute to re-establishing a compensated state within individual compartments and across the integrated pipeline.

**Figure 2.**
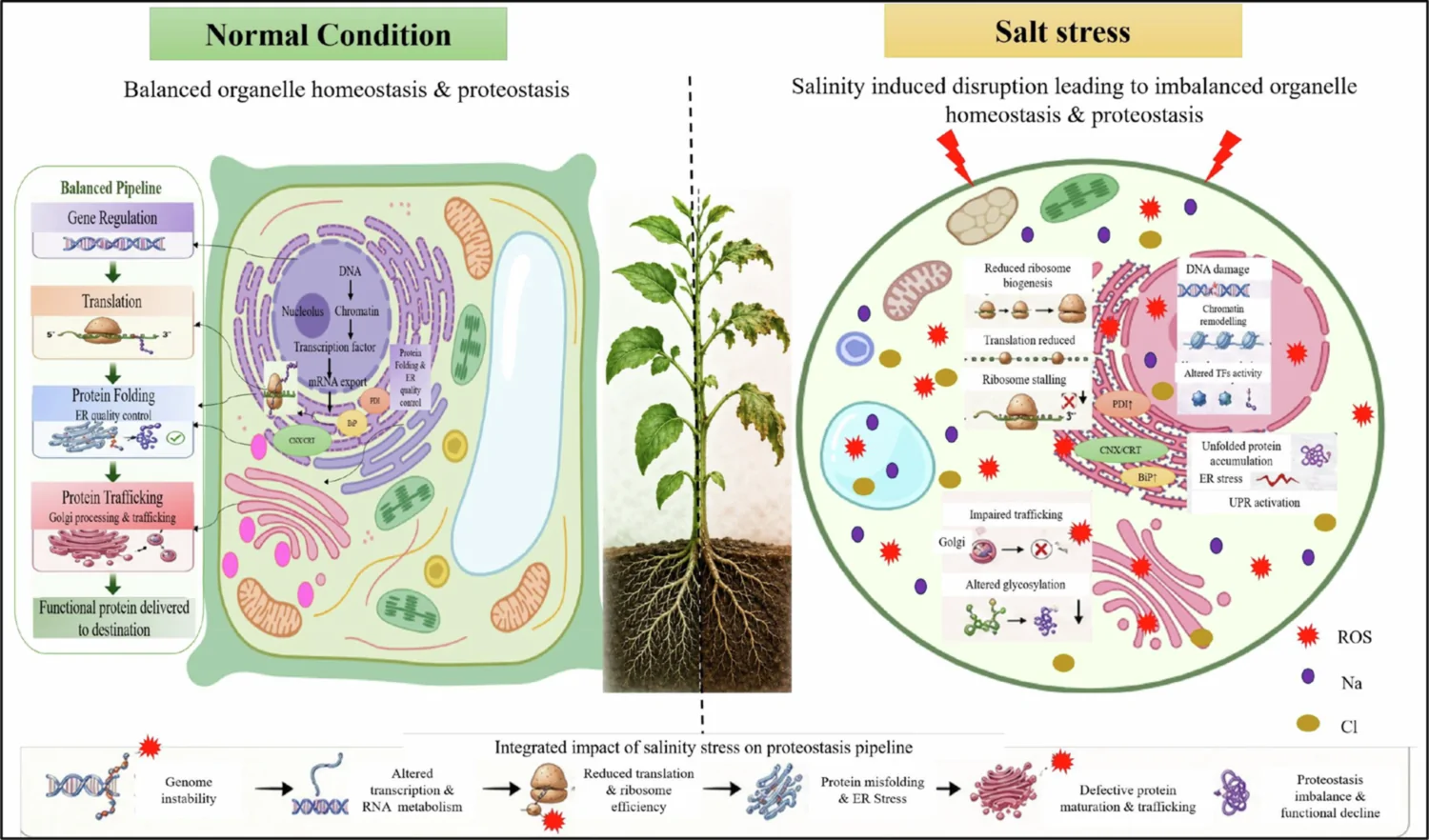
Organelle homeostasis and the proteostasis pipeline under normal conditions versus salinity stress. Under normal conditions, gene regulation, translation, protein folding, and trafficking operate in a balanced pipeline; under salinity stress, disruption at each stage (genome instability, altered transcription, reduced translation, ER stress and protein misfolding, and defective trafficking) cumulatively drives proteostasis imbalance and functional decline.

**Table 1.** Summary of the structural and functional roles, salinity-induced disruptions, restorative mechanisms, and key supporting references for the nucleus, ribosome, endoplasmic reticulum, and Golgi apparatus in plant proteostasis.

Organelle	Key roles under normal conditions	Major salinity-induced disruption	Restorative/compensatory response	References
**Nucleus**	Genome regulation and chromatin organization; nucleolar rRNA transcription and ribosome biogenesis; mRNA processing, editing, and export	Chromatin remodeling and altered histone marks (H3K9/H3K27 acetylation, H3K4me3/H3K27me3); changes in CG/CHG/CHH DNA methylation; nucleolar stress; oxidative damage to DNA, RNA, and nuclear proteins	Activation of DREB, NAC, WRKY, MYB, and bZIP transcription factors; induction of chaperones, antioxidant defenses, and protein quality-control pathways; RNA-binding proteins safeguard transcript processing; HSPs and the ubiquitin–proteasome system restore nuclear proteostasis	Song et al. [55]; Bhadouriya et al. [69]; Hossain et al. [74]; Zhang et al. [34]; Talarico et al. [76,77]; Alhabsi et al. [82]; Wang et al. [35,84]; Yang et al. [88]; Sultana et al. [91]
**Ribosome**	mRNA translation through initiation, elongation, termination, and recycling; nucleolus-dependent 80S cytosolic ribosome biogenesis; 70S ribosomes in chloroplasts and mitochondria	Reduced global translation initiation and impaired rRNA processing and ribosome assembly; oxidative damage to rRNA and ribosomal proteins; ribosome stalling and reduced protein-synthesis capacity	Selective translation of stress-protective mRNAs through translational reprogramming and potentially specialized ribosomes; ribosome-associated quality control (RQC) clears stalled products; ribophagy and molecular chaperones remove or protect damaged translational machinery	Fakih et al. [92]; Suhorukova et al. [38]; Fakih & Germain [37]; Aspden et al. [100]; Xiang & Dong [103]; Gibbs [95]; Rzadkiewicz et al. [96]; Yu et al. [112]; Chen et al. [114]
**Endoplasmic reticulum**	Co-translational protein folding, disulfide-bond formation, and glycosylation mediated by BiP, CNX/CRT, and PDI; ER quality control and COPII-mediated export to the Golgi	Accumulation of unfolded and misfolded proteins; ER stress; disturbed ER–plasma-membrane contact sites; altered redox balance and Ca²^+^ homeostasis; impaired glycoprotein maturation	Activation of the unfolded protein response (IRE1–bZIP60 and bZIP17/28 pathways) increases folding capacity and adjusts protein synthesis; ER-associated degradation (ERAD) removes terminally misfolded proteins; chaperones and antioxidant systems restore ER proteostasis	Howell [124]; Chen et al. [43]; Manghwar & Li [42]; Kim et al. [125]; Christianson et al. [119]; Wang et al. [123]; Ko & Brandizzi [128]; Guay et al. [117]; Rana et al. [129]
**Golgi apparatus**	Glycan maturation, protein processing and sorting, and vesicular trafficking to the plasma membrane, vacuole, cell wall, and extracellular space; synthesis and secretion of cell-wall polysaccharides	Impaired vesicular trafficking and cargo sorting; altered glycosylation; oxidative damage to trafficking proteins and membranes; disruption of cell-wall component delivery and secretory flux	Upregulation of glycosyltransferases and cell-wall-remodeling enzymes; maintenance or remodeling of COPI/COPII- and SNARE-mediated trafficking; ER–Golgi quality-control coordination retrieves or redirects improperly processed proteins	Robinson [133]; Zhang & Zabotina [142]; Dabravolski & Isayenkov [45]; Nagashima et al. [143]; Dünser & Schoberer [140]; Tariq et al. [47]; De Caroli et al. [136]

### 2.5. Post-translational modifications as a rapid layer of proteostasis control

Transcriptional and translational reprogramming act over minutes to hours. Post-translational modifications (PTMs) act more rapidly. They can alter protein activity, stability, localization, or interactions without requiring new protein synthesis. PTMs can therefore contribute to the initial response to salt-induced protein stress before organelle-level programs take effect.^147^ Because PTMs are covalent marks placed directly on the proteome, they also provide a biochemical “alphabet” that links nuclear, ribosomal, ER, and Golgi responses into a coordinated signaling network rather than functioning as a separate regulatory layer.

#### 2.5.1. Phosphorylation and calcium-dependent signaling

Phosphorylation is the most extensively characterized salinity-responsive PTM. Salt-induced Ca^2^^+^ transients activate the well-characterized SOS3-SOS2 kinase module. This module phosphorylates the plasma-membrane Na^+^/H^+^ antiporter SOS1 and promotes Na^+^ efflux. In parallel, ABA-dependent SnRK2 kinases are released from PP2C-mediated inhibition. They phosphorylate downstream transcription factors and ion channels. These processes directly connect PTM regulation with ABA signaling. Phosphoproteomic studies of salt-stressed crops show that many phosphorylation sites are conserved across species. However, the extent of phosphorylation at individual sites can differ between tolerant and sensitive germplasm. In foxtail millet (*Setaria italica*), the more salt-tolerant cultivar Yugu2 shows a faster and more extensive phosphorylation response than the less tolerant cultivar An04 under the same salt treatment. This difference is particularly evident in proteins involved in sucrose metabolism.^148^

#### 2.5.2. Ubiquitination and SUMOylation

Ubiquitination and the structurally related SUMOylation pathway regulate the stability and activity of transcription factors, ion transporters, and quality-control substrates.^149^ In the salt-tolerant sugar beet (*Beta vulgaris*) monosomic addition line M14, comparative ubiquitome profiling identified several hundred ubiquitinated proteins. These proteins were involved in carbohydrate metabolism, photosynthesis, and stress signaling. Their ubiquitination patterns changed within minutes to hours of NaCl exposure.^150^ These findings demonstrate that ubiquitin-mediated proteolysis is an early and substantial component of the crop's salt response. It is not merely a late-stage protein clearance mechanism. Similarly, SUMOylation of stress-responsive transcription factors and chromatin proteins modulates their activity and turnover. This provides an additional layer of regulation. In some cases, SUMOylation can act antagonistically to ubiquitination, thereby influencing the fate and function of the same protein substrates.^149^

#### 2.5.3. Redox- and nitrosative/sulfide-based modifications

Beyond phosphorylation and ubiquitin-like modifications, salinity-induced ROS, RNS, and reactive sulfur species directly modify cysteine residues. These modifications include oxidation, S-nitrosylation, and persulfidation. They are reversible and regulated by thioredoxin. These modifications are also functionally interconnected. In the presence of hydrogen sulfide (H_2_S), S-nitrosylated cysteines can be converted into persulfidated cysteines. Thioredoxin can revert both modifications to the free thiol state. This creates a shared redox “switchboard” that connects nuclear, ribosomal, and ER quality-control systems.^151^ ^,^ ^152^ In sunflower (*Helianthus annuus*) seedlings, salt stress induces opposite, tissue-specific patterns of protein S-nitrosylation. S-nitrosylation increases in cotyledons but decreases in roots. This indicates that the modification is spatially regulated rather than a uniform response to stress.^153^ In tomato, exogenous nitric oxide donors increase protein S-nitrosylation and improve salt tolerance.^130^ Under normal conditions, approximately one in twenty Arabidopsis proteins is persulfidated. Persulfidation levels increase further under oxidative stress. This modification has been proposed to protect cysteine residues against irreversible thiol oxidation.^151^

### 2.6. Phytohormone and gasotransmitter signaling in proteostasis regulation

Organelle-specific responses are not regulated by gene products alone. Small, diffusible signals also modulate these responses. These include phytohormones and gasotransmitters. They integrate information across the cell. In several cases, they also act directly on protein quality-control machinery.

#### 2.6.1. Abscisic acid as the central integrating hormone

Abscisic acid (ABA) accumulates rapidly under salinity. It activates the well-established PYR/PYL/RCAR–PP2C–SnRK2 signaling module. This pathway regulates transcription factors and ion-transport proteins through phosphorylation. These proteins contribute to nuclear responses and Golgi-mediated trafficking. ABA signaling is also regulated by PTMs. In guard cells, H_2_S persulfidates the negative regulator SnRK2.6. This modification increases the sensitivity of ABA signaling. It also links gasotransmitter signaling directly to the phosphorylation network^43^.

#### 2.6.2. Nitric oxide

Nitric oxide (NO) acts largely through S-nitrosylation of target proteins and the formation of S-nitrosoglutathione (GSNO). GSNO is a mobile reservoir of NO. Its turnover is regulated by GSNO reductase (GSNOR). Salt stress alters GSNOR activity and the S-nitroso-proteome in a tissue-specific manner. Changes in NO availability can directly influence salinity tolerance in crop seedlings. In tomato, GSNO pretreatment increases antioxidant capacity and osmolyte accumulation. It also improves growth under NaCl stress.^130^ ^,^ ^153^

#### 2.6.3. Hydrogen sulfide

Hydrogen sulfide (H₂S) signals mainly through cysteine persulfidation. It also interacts closely with NO. Both molecules can target the same cysteine residues. Their modifications can also be reversed through a common thioredoxin-dependent mechanism.^152^ ^,^ ^154^ H₂S also plays a role in ABA signaling. In addition, it enhances antioxidant enzyme activity and promotes osmolyte accumulation under salinity in several plant species. Thus, H₂S can act both as a direct protein modifier and as an upstream regulator of antioxidant defenses. These defenses help protect cellular proteins from oxidative damage under salt stress.^152^

#### 2.6.4. Carbon monoxide

Carbon monoxide (CO), generated endogenously by heme oxygenase (HO), is a less extensively studied but mechanistically distinct gasotransmitter. In sorghum (*Sorghum bicolor*) seedlings, an exogenous CO donor increases HO activity, raises proline and protein content, restores vascular-bundle structure, and lowers the Na^+^/K^+^ ratio and H₂O₂ accumulation under salt stress, while an HO inhibitor and a CO scavenger abolish this protection, directly demonstrating a causal, HO-CO-dependent tolerance pathway in a crop species.^155^ CO signaling converges with NO and H₂S on antioxidant gene expression and cross-talks with ABA, IAA, and gibberellin signaling, reinforcing that the three gasotransmitters act as a coordinated, rather than redundant, signaling module.^155^ ^,^ ^156^

#### 2.6.5. Melatonin as an upstream coordinator

Melatonin integrates phytohormone and gasotransmitter signals with antioxidant and proteostasis responses. These responses involve the four core organelles. Salt stress and exogenous melatonin can increase endogenous melatonin levels. This increase is partly mediated by the phyto-melatonin receptor CAND2/PMTR1. Melatonin then activates downstream NO and polyamine signaling. It also regulates gene expression related to antioxidant defense and ion homeostasis.^157^ Melatonin also interacts with NO, H₂S, and CO. It is further connected with several PTM pathways. Changes in ROS and gasotransmitter levels induced by melatonin can influence the PTM of target proteins. Thus, melatonin may function as a coordinating signal within the salinity-proteostasis network rather than acting only as a protective molecule.^158^ Melatonin, NO, H₂S, and CO can converge on overlapping antioxidant and PTM targets. Their actions are therefore interconnected rather than completely independent. Together, they form a small-molecule signaling layer that influences transcription, translation, protein folding, and vesicle trafficking. Table 2 summarizes representative transcription factors, receptors, and other regulatory proteins involved in this integrated network across the species discussed in this review.

**Table 2.** Representative molecular components (transcription factors, receptor kinases, and other regulatory proteins) linking salinity signaling to protein homeostasis across plant species.

Category	Molecular component	Plant species studied	Role in salinity-associated proteostasis signaling	References
**Transcription factor**	DREB2A	Arabidopsis, rice	Activates osmotic-stress and chaperone-gene expression downstream of nuclear chromatin remodeling	Xiong et al. [78]
**Transcription factor**	NAC family	Wheat, rice, Arabidopsis	Regulates ion-transport and antioxidant genes; integrates with epigenetic marks under salinity	Zheng et al. [79]; Ahmed et al. [25]
**Transcription factor**	WRKY75	Arabidopsis	Positive regulator of salt tolerance; represses the DUF247 gene *SR3G*, linking WRKY signaling to root suberization and Na^+^ accumulation	Rahmati Ishka et al. [159]
**Transcription factor**	bZIP17/bZIP28/bZIP60	Arabidopsis, rice	ER-to-nucleus UPR signaling; relocate from ER/Golgi to nucleus to induce chaperone genes	Ko & Brandizzi [128]; Manghwar & Li [42]
**Nucleolar protein**	APUM23 (Pumilio-domain)	Arabidopsis	Loss-of-function destabilizes rRNA processing and confers ABA-hypersensitive salt sensitivity	Huang et al. [81]
**Receptor-like kinase**	FERONIA (FER)/RALF ligands	Arabidopsis, quinoa	Senses salt-induced cell-wall softening via a malectin-like domain; required for post-stress root growth recovery; family expanded in a halophytic crop	Feng et al. [145]; Jiang et al. [146]
**Ion transporter/regulator**	SOS1/SOS2/SOS3 module	Arabidopsis, Salicornia	Ca²^+^-activated kinase cascade phosphorylates the Na^+^/H^+^ antiporter SOS1; SOS1 shows tonoplast neo-localization in an extreme halophyte	Salazar et al. [107]
**Intrinsically disordered protein**	SALTY (RGG-domain)	Salicornia	Binds ribosomes and RNA-containing structures, stabilizing translation under extreme salinity; no ortholog in glycophyte models	Salazar et al. [107]
**Ion transport-regulatory protein**	Cytochrome b5 (OsCYB5-2)/OsHAK21	Rice	ER-resident post-translational tuning of a plasma-membrane K^+^ transporter	Song et al. [131]
**Chaperone**	BiP/HSP70/HSP90	Multiple species (crop and model)	Expanded under salinity to increase ER and cytosolic folding capacity; expression level correlates with tolerance	Manghwar & Li [42]; Yang et al. [88]
**PTM enzyme/target**	SnRK2.6 (H₂S-persulfidated)	Arabidopsis (guard cells)	Persulfidation sensitizes ABA signaling, linking gasotransmitter and phosphorylation networks	Chen et al. [160]
**Signaling enzyme**	Heme oxygenase (HO1)	Sorghum	Generates the gasotransmitter CO; CO donor restores protein content and ion balance under salt stress	Ikebudu et al. [155]

## 3. Mechanistic crosstalk among the nucleus, ribosome, endoplasmic reticulum, and Golgi apparatus during salinity stress

The preceding sections show that each organelle mounts its own response to salinity. But, these responses do not occur in isolation. Protein modulators, molecular chaperones, PDIs, HSPs, ubiquitin ligases, autophagy components, and UPR regulators physically and functionally connect the nucleus, ribosome, ER, and Golgi. Together, these components form an integrated proteostasis circuit rather than four independent stress-sensing systems.^88^ ^,^ ^129^ This crosstalk is most clearly characterized at the ER-nucleus and ER-Golgi interfaces.^161^ Figure 3 summarizes these interactions. Post-translational modifications and phytohormone/gasotransmitter signals provide the molecular basis for this crosstalk. Phosphorylation, ubiquitination, and cysteine-based redox modifications directly regulate the chaperones, transcription factors, and transporters involved in these responses. Meanwhile, ABA, NO, H₂S, CO, and melatonin act as diffusible signals. They help coordinate the timing of responses across cellular compartments.

**Figure 3.**
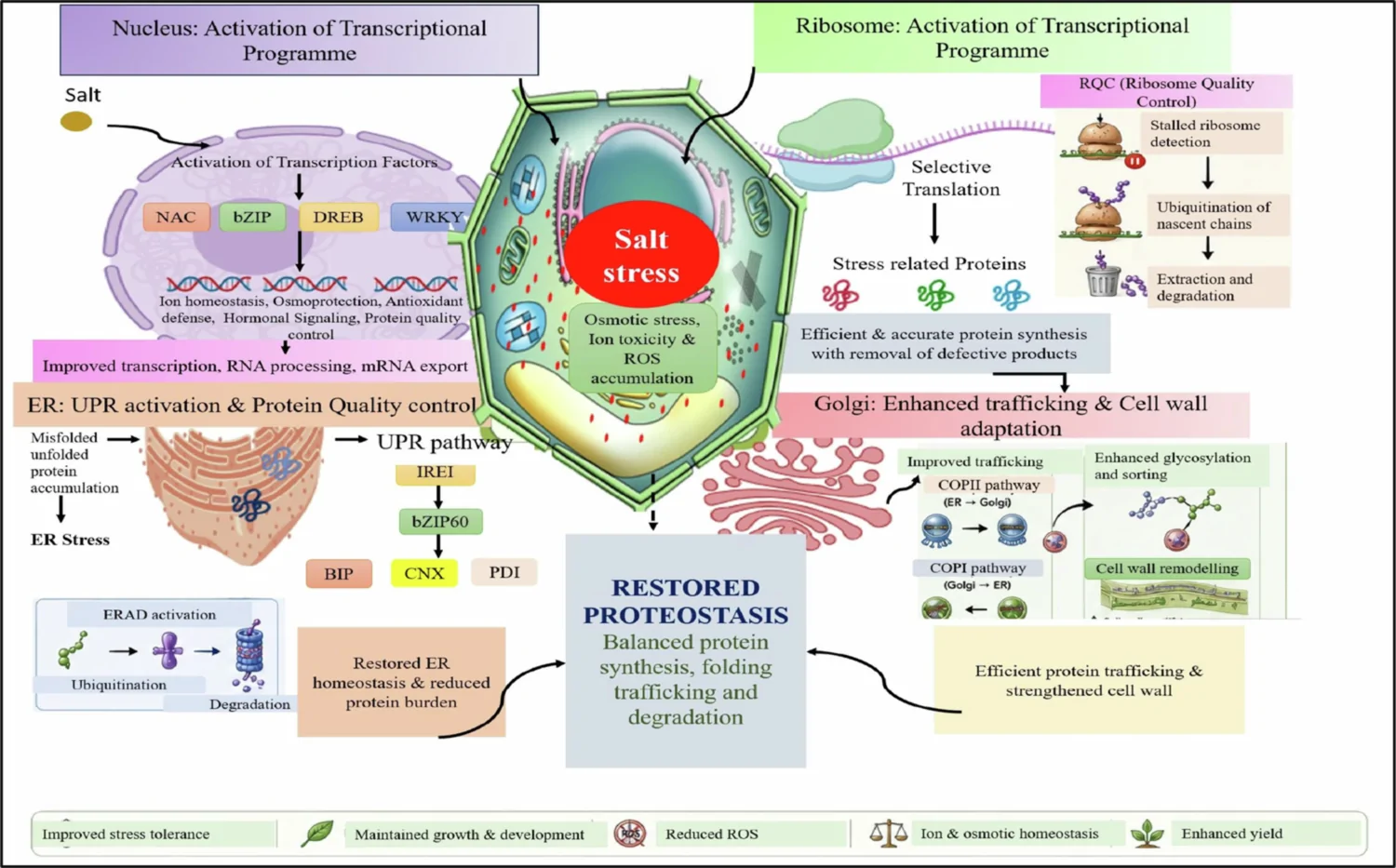
Mechanistic crosstalk among the nucleus, ribosome, endoplasmic reticulum, and Golgi apparatus during salinity stress. Salt stress triggers activation of the transcriptional program in the nucleus, selective translation of stress-related proteins by the ribosome coupled with ribosome-associated quality control (RQC), UPR activation and protein quality control in the ER, and enhanced trafficking and cell-wall adaptation via the Golgi; these organelle-specific responses converge to restore proteostasis (balanced protein synthesis, folding, trafficking, and degradation) and support improved stress tolerance.

The clearest example is UPR-mediated ER-to-nucleus signaling. Upon salt-induced accumulation of misfolded proteins, ER-resident bZIP17 and bZIP28 relocate to the Golgi for proteolytic processing and, together with the IRE1-spliced form of bZIP60, translocate into the nucleus, where they activate genes encoding chaperones, ERAD components, and antioxidant enzymes - closing a feedback loop in which ER protein-folding status directly reprograms nuclear transcription.^42^ ^,^ ^128^ ^,^ ^129^ Nuclear ubiquitin ligases reciprocally regulate the stability of these and other stress-responsive transcription factors, allowing rapid, reversible remodeling of the transcriptome as salt exposure fluctuates.^90^

At the ribosome, newly emerging polypeptides are met immediately by ribosome-associated chaperones (HSP70, HSP40, the nascent-polypeptide-associated complex) that prevent aggregation of nascent chains, while RQC machinery removes products of stalled, salt-damaged ribosomes before they can burden downstream folding capacity in the ER^95^; this coupling means that translational quality control effectively pre-screens the protein load the ER must handle. Correctly folded ER cargo is then exported to the Golgi via COPII vesicles, while COPI vesicles recycle ER-resident chaperones back from the Golgi, and within the Golgi the same protein-modulator network oversees glycosylation and quality control of membrane transporters and receptor kinases - including the Na^+^/H^+^ antiporters, aquaporins, and ATPases whose correct delivery to the plasma membrane is required for ion homeostasis under salt stress before their release to the plasma membrane or vacuole.

This four-organelle relay is not merely additive: because each checkpoint (ribosome, ER, Golgi) filters the protein load reaching the next, and because ER/Golgi stress signals feedback to reprogram nuclear transcription, disruption at any single node under salinity propagates system-wide, while efficient coordination across all four compartments, rather than robustness of any one alone, is what distinguishes salt-tolerant from salt-sensitive genotypes.^88^ ^,^ ^114^ Comparative evidence for this system-level distinction remains limited, and directly contrasting the coordination efficiency of this nucleus-ribosome-ER-Golgi relay between tolerant and sensitive genotypes of the same species is an important open question for future multi-omics studies.

## 4. Future perspectives

Several knowledge gaps limit the translation of the mechanisms reviewed here into practical applications. First, most mechanistic evidence comes from Arabidopsis. Comparable data from major crop species remain limited. Second, studies typically examine one organelle or pathway at a time. Direct, real-time evidence of communication among the nucleus, ribosome, ER, and Golgi during salinity stress remains scarce. For example, live-cell imaging of the ER-to-nucleus UPR relay as it unfolds is still limited. Third, the structural and regulatory basis of specialized or heterogeneous ribosomes remains incompletely understood. The extent to which ER-Golgi quality-control coordination differs between salt-tolerant and salt-sensitive genotypes is also unclear. Fourth, most datasets consist of transcriptomic or proteomic snapshots. Integrated, time-resolved multi-omics studies remain limited. Functional validation under field conditions, rather than controlled environments, is also scarce. Fifth, the post-translational modifications and gasotransmitter signals have mostly been investigated individually. It remains largely unknown how phosphorylation, ubiquitination, S-nitrosylation, and persulfidation are prioritized or combined on the same substrate protein during salt stress. The crop species in which these interactions occur also require further investigation.

Closing these knowledge gaps will require investigating responses across multiple fuctional organization and developmental stages, as salinity tolerance is a highly complex trait governed by mechanisms acting across spatial and temporal scales.^162^ Single-cell transcriptomics and translatomics can reveal cell-type-specific responses. Spatial proteomics, phosphoproteomics, and glycoproteomics can further resolve organelle-specific proteostasis dynamics. These approaches will be needed to capture changes that bulk-tissue assays may overlook. Furthermore, advanced imaging techniques can also help investigate inter-organelle communication. Cryo-electron microscopy, super-resolution microscopy, and live-cell imaging can visualize these interactions directly. For example, they can track ER-nucleus bZIP60/28/17 shuttling and ER-Golgi vesicle exchange during salt exposure. This would provide direct evidence of these processes rather than relying on static experimental endpoints. AI-guided proteomic and structural analyzes can help identify potential regulatory proteins. Machine-learning approaches can integrate multi-omics datasets and prioritize candidate regulators of the nucleus-ribosome-ER-Golgi network for functional testing. CRISPR/Cas-based genome editing and synthetic-biology approaches can then be used to validate and manipulate these candidates. Finally, these approaches must be extended to economically important crop species. Candidate genes and regulatory mechanisms should also be validated under field-realistic salinity conditions. Such efforts will be essential for translating the organelle-crosstalk framework proposed in this review into the development of salinity-resilient crop varieties.

## 5. Conclusion

Salt stress simultaneously affects the nucleus, ribosome, ER, and Golgi apparatus. These disturbances arise from combined osmotic, ionic, oxidative, and metabolic imbalances. But, none of these organelles responds or recovers in isolation. The nucleus reprograms transcription and chromatin states. Ribosomes selectively translate stress-responsive proteins while maintaining translational fidelity. The ER increases its protein-folding capacity through the UPR. It also removes misfolded proteins through ERAD. Meanwhile, the Golgi maintains glycosylation, protein sorting, and secretory delivery. These functions ensure the proper localization of ion transporters required for osmotic and ionic homeostasis. These four compartments are physically and functionally interconnected. As detailed in section 3, they share a network of chaperones, the ubiquitin-proteasome system, RQC, ERAD, autophagy, and vesicle trafficking. Disruption at any node can therefore affect the entire system. Coordinated repair across all four compartments helps restore proteome function. Post-translational modifications and phytohormone/gasotransmitter signals further coordinate this network. As reviewed in sections 2.5 and 2.6, these signals provide a shared molecular vocabulary. It includes phosphorylation, ubiquitination, redox modifications, and nitrosative and sulfide-related marks. ABA-, NO-, H₂S-, CO-, and melatonin-dependent signaling also contributes to this coordination. Together, these mechanisms allow the four organelles to function as an integrated circuit rather than as independent stress sensors. Protein homeostasis under salinity is therefore not simply a property of an individual organelle. Instead, it emerges from the coordinated activities of the entire cellular network. This integrated perspective shifts the focus of crop improvement beyond individual stress-responsive genes. It highlights the coordination points that connect organelles as potential molecular targets. Systematic testing across salt-tolerant and salt-sensitive crop genotypes will be essential to evaluate this framework in future research.
